# Role of enhanced external counterpulsation in long COVID

**DOI:** 10.1515/med-2025-1216

**Published:** 2025-06-16

**Authors:** Sachin A. Shah, Quy Phan, Joshua A. Radel

**Affiliations:** Thomas J. Long School of Pharmacy, University of the Pacific, Stockton, CA, United States of America; David Grant USAF Medical Center, Travis AFB, CA, United States of America; 1st Special Operations Medical Group, Hurlburt Field, FL, United States of America

**Keywords:** post-acute COVID-19 syndrome, enhanced external counter-pulsation, endothelial dysfunction, fatigue, long COVID, brain fog

To the Editor,

We commend the review by Huang et al. [1] on the role of Enhanced External Counterpulsation (EECP) on Long Covid (LC). Additionally, we provide an updated summary of the landscape since key studies are missing in their review article. Huang et al. [1] examined the theoretical mechanisms of EECP for post-acute sequelae of COVID-19 treatment, while Fox et al. [2,3] provided empirical evidence demonstrating significant symptom improvements following EECP intervention, including objective functional metrics and offered a unique analysis that includes a LC comparator group.

As of June 2024, nearly 47 million (18.3%) adults in the United States have experienced LC with 12 million (4.5%) of them reporting limitations in their everyday activity [4]. LC is generally defined as symptoms persisting for more than 12 weeks after having a COVID-19 infection, and not explained by an alternative diagnosis. While multiple organ systems can be impacted, cardiorespiratory (cardiac abnormalities [58%], shortness of breath [40%]) and neurological symptoms (fatigue [32%], cognitive impairment [22%]) are most common [5]. Though a comprehensive treatment guideline is still lacking, the American Academy of Physical Medicine and Rehabilitation recommends treating specific symptoms separately through risk mitigation strategies and specialist referrals. Emerging therapies for the management of LC have mostly included lifestyle modifications in conjunction with rehabilitation interventions, dietary supplements, and hyperbaric oxygen therapy. The prevailing underlying mechanism entails improving endothelial dysfunction, the likely pathology driving these post-acute sequelae of COVID-19.

EECP is a non-invasive (often considered “passive exercise”) treatment modality indicated for the management of refractory angina and ischemic heart failure. EECP is known to improve major adverse cardiovascular event related endpoints such as the Canadian Cardiovascular Society (CCS) Angina Class, Duke Activity Status Index (DASI), Seattle Angina Questionnaire (SAQ), and the 6 Minute Walk Test (6MWT). During EECP, pneumatic cuffs are wrapped around the calves, thighs, and buttocks, which sequentially inflate from distal to proximal regions in early diastole followed by rapid deflation at the onset of systole. A standard course of EECP treatment consists of 35 1 h sessions over a 7-week period.

In a randomized, sham-controlled trial in patients with symptomatic coronary artery disease, EECP improved endothelial function evident via a 30–50% improvement in peripheral artery flow-mediated dilation and a 171% increase in plasma endothelial-derived vasoactive agents. The potential application of EECP for LC was first validated in 2021, where improvements in shortness of breath, fatigue, and brain fog were noted after 15 h of EECP in a 38-year-old female [6]. Subsequently, a prospective evaluation of ten patients with LC-associated angina and objective evidence of microvascular dysfunction showed significant improvements in angina and fatigue post 15 h of EECP [7]. Improvements in 6MWT were not statistically significant (+29.5 m), likely due to the small sample size [7]. Most recently, a large observational cohort (*n* = 231) looking at EECP in LC patients found significant improvements in SAQ (+19.8 ± 21.1), Patient-Reported Outcomes Measurement Information System (PROMIS) fatigue (−13.2 ± 9.7), DASI (+20.8 ± 14.7), and 6MWT (+151.6 ± 199.1 ft) (all *p*-values <0.001) [2]. Notably, significant improvements were seen in angina and dyspnea burden, and most patients were able to return to work (18 of 23) [2].

Previous studies [2,6,7] regarding EECP use in LC have been limited from the lack of a true control group; however, a small comparison of EECP-treated LC patients compared to a free-living LC cohort has found data to suggest the need for a larger randomized clinical trial. In the study by Fox et al. [2], LC patients (*N* = 33) undergoing EECP were compared to a sex, age, and fatigue severity matched LC participants (*N* = 33), and significant improvements were found in fatigue, angina, and dyspnea [PROMIS Fatigue score (−15.0 ± 8.9 vs −2.8 ± 5.9, *p* < 0.001); DASI (+17.8 (11.8, 26.8) vs +1.8 (−3.5, 5.5), *p* < 0.001); >1 improvement in Rose Dyspnea class (75.8 vs 33.3%, *p* < 0.001)] [2]. Similarly, in another sub-group analysis, LC patients (*N* = 38) with objectively assessed cognitive impairment (BrainCheck score ≤77.5) were compared to a control group without cognitive impairment (*N* = 42), and significant improvements were noted post EECP [8].

Despite the preponderance of data (Table 1) and the clinically meaningful magnitude of benefit, several limitations need to be addressed in future research. Notably, these trials should incorporate larger sample sizes, randomized sham-controlled study designs, and a treatment regimen of 35 1 h EECP sessions to maximize clinical benefits. In the absence of any FDA-approved treatments for LC, EECP appears to be a promising therapeutic option.

**Table 1:** Notable EECP studies assessing LC outcomes

Study	Patient population	Intervention, treatment duration	Control	Notable outcomes
Dayrit et al. [6]	LC (*n* = 1)	EECP, 15 h	None	Dyspnea*
				Fatigue*
				Brain fog*
Wu et al. [7]	LC-associated angina and microvascular dysfunction (*n* = 10)	EECP, 15 h	None	6MWT
				HADS
				RAND36
				Brain Fog*
				Angina Symptoms*
				Fatigue*
				Return to work*
Fox et al. [2]	LC (*n* = 231)	EECP, 25–45 h	None	SAQ
				PROMIS
				DASI
				6MWT
				CCS
				RDS
				Brain Fog*
				Return to work*
Fox et al. [3]^	LC (*n* = 66)	EECP, 25–45 h	Free-living matched control	PROMIS
				DASI
				RDS
Sathyamoorthy et al. [8]^	LC, CI (*n* = 38)	EECP, 25–45 h	EECP, 25–45 h on NCI	BrainCheck Composite Score
	LC, NCI (*n* = 42)			

Abbreviations: EECP, Enhanced External Counterpulsation; LC, Long COVID; 6MWT, Six Minute Walk Test; HADS, Hospital Anxiety and Depression Scale; SAQ, Seattle Angina Questionnaire; PROMIS, Patient-Reported Outcomes Measurement Information System; DASI, Duke Activity Status Index; CCS, Canadian Cardiovascular Society; RDS, Rose Dyspnea Scale; CI, Cognitive impairment; NCI, No cognitive impairment.

*Subjective patient reported outcomes using a non-validated assessment.

^Sub-group analyses/sub-studies of Fox et al. (*n* = 231).
